# TARE-Induced Pan-Immune Inflammation Value as a Prognostic Biomarker in Liver-Dominant Metastatic Colorectal Cancer

**DOI:** 10.3390/jcm14144927

**Published:** 2025-07-11

**Authors:** Bengu Dursun, Burak Demir, Nejat Emre Öksüz, Çiğdem Soydal, Güngör Utkan

**Affiliations:** 1 Department of Medical Oncology, Ankara University Faculty of Medicine, 06100 Ankara, Turkey; nejatemreoksuz@gmail.com (N.E.Ö.); gungorutkan_md@yahoo.com (G.U.); 2 Department of Medical Oncology, Ankara Etlik City Training and Research Hospital, 06010 Ankara, Turkey; 3 Department of Nuclear Medicine, Şanlıurfa Mehmet Akif Inan Education and Research Hospital, 63200 Sanlıurfa, Turkey; 4burakfe@gmail.com; 4 Department of Nuclear Medicine, Ankara University Faculty of Medicine, 06100 Ankara, Turkey; csoydal@yahoo.com

**Keywords:** TARE, pan-immune inflammation, metastatic colon cancer

## Abstract

**Purpose:** Previous studies have reported that blood-based inflammatory markers are associated with prognosis in patients with various solid tumors, including colorectal cancer (CRC). The pan-immune inflammation value (PIV) is a novel prognostic biomarker based on blood count. Here, we aimed to study the association between PIV and survival following transarterial radioembolization (TARE) in patients with liver-dominant metastatic colorectal cancer (CLM). **Methods:** A total of 49 patients with CLM who underwent TARE at the Ankara University Department of Medical Oncology were retrospectively analyzed. The relationship between clinical and laboratory parameters with post-TARE overall survival (OS) was analyzed by multivariate analyses. **Results:** The median age was 60 years and 71.4% of patients had received at least two lines of chemotherapy. The objective response rate (ORR) was 59.1% following TARE. Patients with hepatic response after TARE treatment demonstrated significantly longer survival compared to non-responders (*p*: 0.033). The optimal PIV threshold value was calculated as 629 in ROC analyses. This PIV value had 81% sensitivity and 80% specificity for OS prediction (AUC 0.86; 95% CI: 0.75–0.98, *p* = 0.008). Patients with elevated PIV > 629 had significantly shorter OS (*p* = 0.002). In the multivariate analysis, adjusted for ECOG PS, TARE response, presence of extrahepatic disease, number of chemotherapy lines, CEA levels and post-TARE NLR and PIV, only low PIV level was associated with longer OS (>629 vs. ≤629; HR: 4.87; 95% CI: 1.32–17.92; *p* = 0.017). **Conclusions:** PIV, a blood-based inflammatory score, may reflect the host’s immune response following TARE and serve as a novel predictor of survival.

## 1. Introduction

Colorectal cancer (CRC) ranks as the third most frequently diagnosed malignancy worldwide and represents the second leading cause of cancer-related mortality [1]. The development of metastases remains the most significant challenge in the treatment of CRC. Hepatic metastases are detected in over one-third of patients at CRC diagnosis and approximately half of patients with early-stage tumors experience metastatic spread to the liver during follow-up after curative surgery [2,3]. While hepatic resection is the gold standard in the treatment of colorectal cancer liver metastases (CLM), only 20% of patients are eligible candidates for hepatic resection [4]. In such cases, locoregional non-surgical approaches may provide symptomatic relief and extend survival beyond systemic therapy alone [5]. Transarterial radioembolization (TARE), also known as selective internal radiation therapy (SIRT), is a minimally invasive procedure that has been increasingly used to treat CLM. Integrating TARE with first- or second-line chemotherapy, with or without biological agents, in patients with liver-limited or liver-dominant metastatic colorectal cancer has not demonstrated a survival benefit [6,7,8,9]. As a result, TARE is generally reserved as a salvage treatment for patients with CLM who have become resistant to multiple prior lines of systemic therapy [10].

Despite promising results, some patients exhibit rapid disease progression after TARE, emphasizing the urgent need for predictive markers to identify those most likely to benefit. Given the crucial role of inflammation in CRC pathogenesis and prognosis, TARE-induced inflammation may potentially influence survival [11]. Evaluating the correlation between TARE-induced inflammatory response by blood-based inflammatory markers could provide insights into treatment efficacy and prognosis. Although data are limited, post-TARE blood-based systemic inflammatory markers such as neutrophil–lymphocyte ratio (NLR) and platelet–lymphocyte ratio (PLR) may be associated with survival outcomes, with higher ratios potentially indicating worse prognosis [12].

The pan-immune-inflammation value (PIV), which incorporates all immune-inflammatory cell types in the peripheral blood count, has been investigated as a biomarker of prognosis across various tumor types [13]. The association between PIV levels and clinical outcomes in CLM patients treated with TARE has not yet been investigated. This study primarily aims to assess the relationship between PIV levels and survival in patients with CLM undergoing TARE.

## 2. Materials and Methods

### 2.1. Study Cohort

This retrospective, single-center study included patients with liver-dominant metastatic CRC who were treated with TARE and followed at the Department of Medical Oncology, Ankara University School of Medicine between April 2013 and September 2024. All patients received a single treatment of SIRT using Yttrium-90 (90Y) microspheres. Data collected included baseline demographics, Eastern Cooperative Oncology Group (ECOG) performance status, prior colorectal surgery, primary tumor location, history of liver-directed therapy, presence of extrahepatic metastases, hepatic tumor burden, number of prior chemotherapy lines, use of biologic agents, CEA levels, TARE response, post-treatment NLR and PIV values, as well as survival outcomes.

PIV was calculated using the formula (neutrophil count [10^3^/mL] × platelet count [10^3^/mL] × monocyte count [10^3^/mL])/lymphocyte count [10^3^/mL]. Radiologic response was assessed according to the RECIST (Response Evaluation Criteria in Solid Tumors) guidelines by comparing follow-up imaging to baseline studies. An objective radiologic response (ORR) was defined as either a partial or complete response. Overall survival (OS) was defined as the interval between TARE treatment and either the last follow-up or death. Ethical approval for this study was obtained from the Clinical Research Ethics Committee of Ankara University School of Medicine.

### 2.2. TARE Treatment

Patients who were referred and found eligible for radioembolization underwent thorough pre-procedural evaluation, including hepatic artery perfusion scintigraphy and SPECT/CT following intra-arterial injection of technetium-99m macroaggregated albumin (99mTc-MAA). Following hepatic artery perfusion scintigraphy, treatments are planned with a multi-compartmental dosimetry model in light of the current guidelines. Lung shunt fraction was calculated with lung and liver region-of-interest (ROI) geometric mean on the anterior and posterior planar images. SPECT/CT sequences were used for the tumour and non-tumour liver dose prediction. Radioembolization procedures were scheduled approximately two weeks after the completion of perfusion scintigraphy. The procedure involved selective catheterization of the hepatic artery and administration of Yttrium-90 (Y-90) microspheres, designed to deliver targeted beta radiation to tumor tissue while sparing normal liver parenchyma. Post-procedure, patients were monitored overnight in the hospital for immediate care. Within 24 h, Y-90 PET imaging was performed to confirm microsphere distribution and verify dosimetric accuracy

### 2.3. Statistical Analysis

Descriptive statistics were reported as medians for continuous variables and as frequencies with percentages for categorical variables. The optimal cutoff for PIV was established through receiver operating characteristic (ROC) analysis based on Youden’s J index. This cutoff was then used to classify the cohort into PIV-high and PIV-low groups.

Comparisons between the PIV-high and PIV-low groups were performed using independent samples *t*-tests for continuous variables and Chi-square tests for categorical variables. OS was defined as the time from TARE treatment to the last follow-up or death, while disease-free survival (DFS) was defined as the interval from TARE to disease progression or death. Survival analyses based on the ROC-derived cutoff value were performed using the Kaplan–Meier method. Multivariate analyses were performed using Cox regression, with hazard ratios (HRs) and 95% confidence intervals (CIs) reported. All statistical analyses were conducted using SPSS version 25.0 (IBM Inc., Armonk, NY, USA) and a two-sided *p*-value of <0.05 was considered statistically significant.

## 3. Results

### 3.1. Baseline Characteristics

A total of 49 patients with CLM underwent TARE treatment. None of the patients underwent concurrent administration of chemotherapy during TARE treatment. The cohort’s median age was 60 (57.9–63.7) years and 59.2% of the patients were males. A total of 85.7% of patients had a good performance status (ECOG 0–1) and 83.7% presented with metastatic disease at diagnosis. Hepatic metastases represented the sole site of metastatic disease in 61.2% of patients, while the remainder had limited extrahepatic involvement. At the time of TARE, 69.4% of patients had <20% hepatic disease burden and all the patients had multiple hepatic metastases. Liver-directed treatment had been administered to 32% of patients before TARE, while 71.4% had been treated with two or more lines of cytotoxic chemotherapy. Nearly ninety percent of patients had previously received biological treatment; 40.8% were treated with anti-EGFR (cetuximab) and 77.5% received anti-VEGF (bevacizumab; only one patient had received regorafenib) (Table 1). Post-TARE PIV and NLR values were observed to have increased significantly compared to pre-treatment levels (*p* < 0.001).

**Figure 1:**
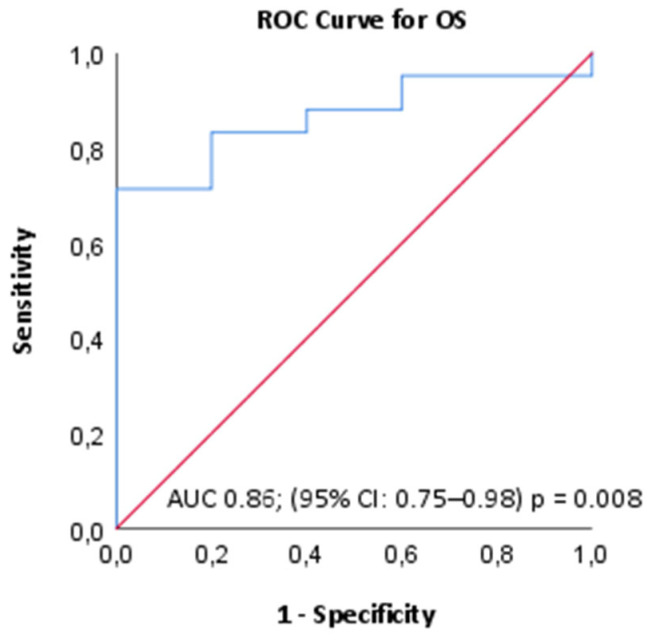
ROC curve analysis of PIV for predicting overall survival.

ROC analysis determined a post-TARE PIV threshold of 629 as the optimal cutoff for predicting OS, yielding a sensitivity of 81% and a specificity of 80% (AUC 0.86; 95% CI: 0.75–0.98; *p* = 0.008) (Figure 1). The patients were dichotomized into the PIV high or low groups according to this PIV value. Patients with higher PIV levels were found to have received more chemotherapy lines and had extrahepatic metastasis (*p*: 0.025 and *p*: 0.017, respectively).

### 3.2. Survival Analyses

After a median of 24 (95% CI: 21.42–26.57) month follow-up, 44 (89.8%) patients died. The median OS from the date of TARE treatment was 10 months (95% CI: 5.5–14.49). The median hepatic PFS was 16 weeks (95% CI: 8.0–24.0).

**Figure 2:**
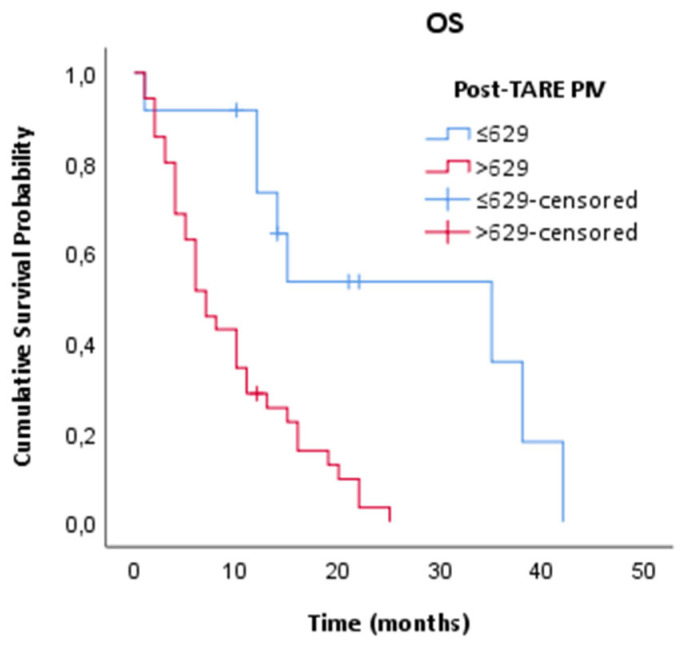
Kaplan–Meier survival curves based on post-TARE PIV classification.

Following TARE, the hepatic objective response rate was 59.1%. Patients who achieved a hepatic response had significantly longer OS compared to non-responders (*p* = 0.001). Pre-TARE NLR and PIV scores were not found to be associated with post-TARE survival (*p*: 0.06 and *p*: 0.08, respectively). Patients with higher post-TARE PIV levels (>629) had lower OS than patients with lower PIV levels (≤629) (*p*: 0.002) (Figure 2). In addition to lower PIV levels, ECOG PS ≤ 1 (*p*: 0.009), low pre-treatment CEA levels (*p*: 0.026), fewer than two lines of chemotherapy (*p*: 0.04) and low post-TARE NLR levels (*p*: 0.011) were associated with longer OS in univariate analysis (Table 2). In the multivariate analysis, only low PIV levels showed significantly increased OS (HR: 4.87, 95% CI: 1.32–17.92; *p* = 0.017) (Table 3).

## 4. Discussion

This study demonstrated that elevated post-TARE PIV serves as an independent prognostic indicator of OS in patients with CLM undergoing TARE. To the best of our knowledge, this is the first study to specifically evaluate the prognostic value of PIV after TARE in mCRC.

Although TARE is increasingly used in CLM, few prognostic markers have been identified. Earlier studies have recognized that extensive hepatic tumor burden, multiple prior chemotherapy regimens, lack of radiologic response to TARE, and presence of extrahepatic disease are the main clinical predictors of OS [10,14]. Consistent with these findings, our study confirmed that patients with good performance status, absence of extrahepatic disease, non-chemoresistant tumors, and low pre-TARE CEA levels showed prolonged OS. Additionally, radiologic response to TARE was associated with significantly longer OS compared to non-responders (15 months vs. 6 months, *p* = 0.033). Apart from radiologic response, these results represent general prognostic indicators in metastatic colorectal cancer rather than factors specifically associated with survival after TARE [15].

The relationship between inflammatory scores and prognosis is complex and remains largely undefined. Both tumor characteristics and host-related factors influence cancer-related survival. The immune response of the host against cancer is largely dependent on lymphocytes, and reduced tumor lymphocytic infiltration is associated with poorer prognosis [16]. As angiogenic factors increase, neutrophil and platelet counts also tend to rise [17]. In the early period after TARE, an acute inflammatory response occurs, leading to epithelial damage, release of cytokines, coagulation factors and modulation of the tumor microenvironment [18,19,20].

Elevated NLR and PIV reflect neutrophilia, monocytosis, thrombocytosis and lymphopenia, each linked to distinct pro-tumorigenic processes such as angiogenesis, suppression of cytotoxic lymphocytes, tumor invasion and metastatic dissemination. The post-TARE setting may amplify these processes, as local radiation-induced inflammation contributes to further alterations in cytokine profiles, recruitment of myeloid-derived suppressor cells and modulation of the tumor microenvironment toward an immune-suppressive state. These mechanistic insights support the notion that PIV not only serves as a marker of systemic inflammation but also encapsulates key immunological dynamics that affect survival following TARE. In contrast, pre-TARE inflammatory scores were not significantly associated with OS in our study, likely because the immune-inflammatory impact of TARE itself plays a dominant role. Consistent with our findings, Young et al. also reported no significant relationship between pre-TARE inflammatory markers and OS following TARE [12].

PIV was developed to enhance the prognostic performance of blood-based inflammatory scores by integrating neutrophils, lymphocytes, monocytes and platelets [21]. A pooled analysis of six studies showed that elevated PIV was significantly associated with reduced OS in colorectal cancer (HR = 2.09; 95% CI: 1.67–2.61; *p* < 0.0001) [22]. Also, in mCRC, elevated PIV predicted poorer OS independent of NLR and platelet levels [21]. In our study, elevated post-TARE NLR and PIV were both linked to reduced OS (*p* = 0.007 and *p* < 0.001, respectively). The association between high PIV and reduced OS may reflect the higher frequency of extrahepatic disease and chemoresistant tumors in this group, while elevated NLR likely represents a parallel inflammatory response contributing to poor outcomes. Importantly, PIV remained independently prognostic even after adjusting for NLR, performance status, extrahepatic disease, prior therapies, CEA levels and radiologic response.

Our findings that elevated post-TARE PIV is linked to poorer survival and more prior chemotherapy raise important questions about the optimal timing of TARE in liver-dominant mCRC. While TARE is currently recommended as a salvage therapy after multiple systemic treatments, it is conceivable that earlier use of TARE, before the development of a pronounced systemic inflammatory response as reflected by high PIV, might improve outcomes. However, given the retrospective design of our study and the uniform use of TARE in the salvage setting, we were unable to directly assess this hypothesis. Prospective studies are needed to explore the therapeutic value of earlier TARE administration in relation to systemic inflammatory status.

These findings support the integration of PIV into future clinical decision-making algorithms. As a simple, cost-effective biomarker, PIV could help identify high-risk patients who may benefit from intensified systemic therapies, closer post-procedural monitoring or inclusion in clinical trials exploring novel immunomodulatory or anti-inflammatory strategies. Furthermore, PIV could be integrated into composite prognostic models alongside clinical and radiological variables to enhance risk stratification and optimize treatment selection. Prospective validation studies are warranted to establish standardized PIV thresholds and to determine the most effective way to implement PIV in routine clinical practice.

The limitations of this study include a limited sample size, heterogeneity of the patient cohort and its retrospective design. Despite these limitations, we identified a significant association between PIV levels and post-TARE survival outcomes irrespective of multiple clinical variables.

## 5. Conclusions

Our study highlights the potential of using PIV score prognostically for survival after TARE in patients with CLM. To confirm these findings and better understand how TARE affects systemic inflammation and survival, future studies with larger cohorts are needed.

## Figures and Tables

**Table 1:** Clinicopathological variables of 49 patients treated with TARE.

Baseline Characteristics	n (%)
Age, median (range), years	60.8 (57.9–63.7)
Sex (Male/Female)	29 (59.2)/20 (40.8)
ECOG PS	
0–1	42 (85.7)
2–3	7 (14.3)
Stage at diagnosis	
Stage 2	2 (4.1)
Stage 3	6 (12.2)
Stage 4	41 (83.7)
Primary tumor location	
Left colon	40 (81.6)
Right colon	9 (18.4)
Primary colorectal surgery	
Yes	30 (61.2)
No	19 (38.8)
Extrahepatic disease	
Yes	19 (38.8)
No	30 (61.2)
Hepatic tumor burden	
≤%20	34 (69.4)
>%20	8 (16.3)
Previous liver-directed therapy	
Yes	16 (32.7)
No	33 (67.3)
Prior chemotherapy lines	
0–1	14 (28.6)
≥2	35 (71.4)
CEA ≥ 5	42 (85.7)

CEA: Carcinoembryonic antigen; ECOG PS: Eastern Cooperative Oncology Group performance status; KRAS: Kirsten rat sarcoma viral oncogene homologue.

**Table 2:** Univariate analysis of factors affecting post-TARE overall survival.

Factors	HR	95% Cl	*p* Value
Age, year (≤60; >60)	0.76	0.40–1.41	0.38
Sex (Male/Female)	1.23	0.65–2.31	0.51
ECOG PS (≤1; >1)	3.05	1.28–7.24	**0.011**
Stage at diagnosis (Non-metastatic; Metastatic)	1.45	0.64–3.30	0.37
Primary tumor location (Left; Right)	1.36	0.63–2.95	0.43
Primary colorectal surgery (Yes/No)	0.67	0.36–1.27	0.22
Extrahepatic disease (Yes/No)	0.53	0.29–0.99	**0.049**
Hepatic tumor burden (≤%20; >%20)	1.08	0.49–2.36	0.84
Previous liver-directed therapy (Yes/No)	0.65	0.32–1.30	0.22
Prior chemotherapy lines (0–1; ≥2)	2.18	1.03–4.48	**0.04**
Anti-VEGF before TARE (Yes/No)	1.48	0.73–3.02	0.27
Anti-EGFR before TARE (Yes/No)	0.68	0.36–1.28	0.23
Pretreatment CEA (≤/>5)	2.65	1.01–6.91	**0.046**
TARE response (+/−)	2.00	1.02–3.91	**0.042**
Post-TARE NLR (≤/>5)	2.50	1.23–5.09	**0.011**
Post-TARE PIV (≤/>629)	4.61	1.77–12.0	**0.002**

The bolded values represent the statistically significant values. CEA: Carcinoembryonic antigen; ECOG PS: Eastern Cooperative Oncology Group performance status; EGFR: Epithelial growth factor; VEGF: Vascular endothelial growth factor; NLR: Neutrophil/lymphocyte ratio; PIV: Pan-immune inflammation.

**Table 3:** Multivariate analysis of factors associated with survival following TARE.

Factors	HR	95% Cl	*p* Value
ECOG PS (≤1; >1)	2.24	0.74–6.79	0.15
Extrahepatic disease (Yes/No)	0.72	0.29–1.78	0.48
Prior chemotherapy lines (0–1; ≥2)	1.27	0.48–3.36	0.62
Pre-treatment CEA (≤5/>5)	2.08	0.71–6.06	0.17
TARE response	1.86	0.85–4.10	0.12
Post-TARE NLR (≤/>5)	1.17	0.50–2.70	0.70
Post-TARE PIV (≤/>629)	4.87	1.32–17.92	0.017

CEA: Carcinoembryonic antigen; ECOG PS: Eastern Cooperative Oncology Group performance status; HR: Hazard ratio; NLR: Neutrophil/lymphocyte ratio; PIV: Pan-immune inflammation.

## Data Availability

The data generated or analyzed in this study can be obtained from the corresponding author upon reasonable request.

## References

[1] Siegel R.L., Miller K.D., Wagle N.S., Jemal A. (2023). Cancer statistics, 2023. CA Cancer J. Clin..

[2] van der Geest L.G., Lam-Boer J., Koopman M., Verhoef C., Elferink M.A., de Wilt J.H. (2015). Nationwide trends in incidence, treatment and survival of colorectal cancer patients with synchronous metastases. Clin. Exp. Metastasis.

[3] van Gestel Y.R., de Hingh I.H., van Herk-Sukel M.P., van Erning F.N., Beerepoot L.V., Wijsman J.H., Slooter G.D., Rutten H.J.T., Creemers G.-J.M., Lemmens V.E.P.P. (2014). Patterns of metachronous metastases after curative treatment of colorectal cancer. Cancer Epidemiol..

[4] Adam R., Vinet E. (2004). Regional treatment of metastasis: Surgery of colorectal liver metastases. Ann. Oncol..

[5] Uhlig J., Lukovic J., Dawson L.A., Patel R.A., Cavnar M.J., Kim H.S. (2021). Locoregional therapies for colorectal cancer liver metastases: Options beyond resection. Am. Soc. Clin. Oncol. Educ. Book..

[6] Sharma R.A., Van Hazel G.A., Morgan B., Berry D.P., Blanshard K., Price D., Bower G., Shannon J.A., Gibbs P., Steward W.P. (2007). Radioembolization of liver metastases from colorectal cancer using yttrium-90 microspheres with concomitant systemic oxaliplatin, fluorouracil, and leucovorin chemotherapy. J. Clin. Oncol..

[7] Van Hazel G.A., Heinemann V., Sharma N.K., Findlay M.P., Ricke J., Peeters M., Perez D., Robinson B.A., Strickland A.H., Ferguson T. (2016). SIRFLOX: Randomized phase III trial comparing first-line mFOLFOX6 (plus or minus bevacizumab) versus mFOLFOX6 (plus or minus bevacizumab) plus selective internal radiation therapy in patients with metastatic colorectal cancer. J. Clin. Oncol..

[8] Wasan H.S., Gibbs P., Sharma N.K., Taieb J., Heinemann V., Ricke J., Peeters M., Findlay M., Weave A., Mills J. (2017). First-line selective internal radiotherapy plus chemotherapy versus chemotherapy alone in patients with liver metastases from colorectal cancer (FOXFIRE, SIRFLOX, and FOXFIRE-Global): A combined analysis of three multicentre, randomised, phase 3 trials. Lancet Oncol..

[9] Mulcahy M.F., Mahvash A., Pracht M., Montazeri A.H., Bandula S., Martin R.C., Herrmann K., Brown E., Zuckerman D., Wilson G. (2021). Radioembolization with chemotherapy for colorectal liver metastases: A randomized, open-label, international, multicenter, phase III trial. J. Clin. Oncol..

[10] Saxena A., Bester L., Shan L., Perera M., Gibbs P., Meteling B., Morris D.L. (2014). A systematic review on the safety and efficacy of yttrium-90 radioembolization for unresectable, chemorefractory colorectal cancer liver metastases. J. Cancer Res. Clin. Oncol..

[11] Yamamoto T., Kawada K., Obama K. (2021). Inflammation-related biomarkers for the prediction of prognosis in colorectal cancer patients. Int. J. Mol. Sci..

[12] Young S., Ragulojan R., Todatry S., D’Souza D., Golzarian J., Flanagan S., Sanghvi T. (2023). Evaluation of inflammatory scores in metastatic colorectal cancer patients undergoing transarterial radioembolization. Cardiovasc. Interv. Radiol..

[13] Guven D.C., Sahin T.K., Erul E., Kilickap S., Gambichler T., Aksoy S. (2022). The association between the pan-immune-inflammation value and cancer prognosis: A systematic review and meta-analysis. Cancers.

[14] Saxena A., Meteling B., Kapoor J., Golani S., Morris D.L., Bester L. (2015). Is yttrium-90 radioembolization a viable treatment option for unresectable, chemorefractory colorectal cancer liver metastases? A large single-center experience of 302 patients. Ann. Surg. Oncol..

[15] Stillwell A.P., Ho Y.-H., Veitch C. (2011). Systematic review of prognostic factors related to overall survival in patients with stage, I.V. colorectal cancer and unresectable metastases. World J. Surg..

[16] Okano K., Maeba T., Moroguchi A., Ishimura K., Karasawa Y., Izuishi K., Goda F., Usuki H., Wakabayashi H., Maeta H. (2003). Lymphocytic infiltration surrounding liver metastases from colorectal cancer. J. Surg. Oncol..

[17] Kusumanto Y.H., Dam W.A., Hospers G.A., Meijer C., Mulder N.H. (2003). Platelets and granulocytes, in particular the neutrophils, form important compartments for circulating vascular endothelial growth factor. Angiogenesis.

[18] Fernandez-Ros N., Iñarrairaegui M., Paramo J.A., Berasain C., Avila M.A., Chopitea A., Varo N., Sarobe P., Bilbao J.I., Dominguez I. (2015). Radioembolization of hepatocellular carcinoma activates liver regeneration, induces inflammation and endothelial stress and activates coagulation. Liver Int..

[19] Seidensticker M., Powerski M., Seidensticker R., Damm R., Mohnike K., Garlipp B., Klopffleisch M., Amthauer H., Ricke J., Pech M. (2017). Cytokines and 90 Y-radioembolization: Relation to liver function and overall survival. Cardiovasc. Interv. Radiol..

[20] Soydal Ç., Araz M., Durmaz M., Özkan E., Ergüder B.İ., Küçük N.Ö., Bilgiç S., Elhan A.H., Geçim I.E. (2022). Elevated Angiogenic Factor Levels After Transarterial Radioembolization for Colorectal Cancer Liver Metastases May Predict a Poor Prognosis. Mol. Imaging Radionucl. Ther..

[21] Fucà G., Guarini V., Antoniotti C., Morano F., Moretto R., Corallo S., Marmorino F., Lonardi S., Rimassa L., Sartore-Bianchi A. (2020). The Pan-Immune-Inflammation Value is a new prognostic biomarker in metastatic colorectal cancer: Results from a pooled-analysis of the Valentino and TRIBE first-line trials. Br. J. Cancer.

[22] Yang X.-C., Liu H., Liu D.-C., Tong C., Liang X.-W., Chen R.-H. (2022). Prognostic value of pan-immune-inflammation value in colorectal cancer patients: A systematic review and meta-analysis. Front. Oncol..

