# Correction: Dursun et al. TARE-Induced Pan-Immune Inflammation Value as a Prognostic Biomarker in Liver-Dominant Metastatic Colorectal Cancer. *J. Clin. Med.* 2025, *14*, 4927

**DOI:** 10.3390/jcm14228218

**Published:** 2025-11-20

**Authors:** Bengu Dursun, Burak Demir, Nejat Emre Öksüz, Çiğdem Soydal, Güngör Utkan

**Affiliations:** 1 Department of Medical Oncology, Ankara University Faculty of Medicine, 06100 Ankara, Turkey; nejatemreoksuz@gmail.com (N.E.Ö.); gungorutkan_md@yahoo.com (G.U.); 2 Department of Medical Oncology, Ankara Etlik City Training and Research Hospital, 06010 Ankara, Turkey; 3 Department of Nuclear Medicine, Şanlıurfa Mehmet Akif Inan Education and Research Hospital, 63200 Sanlıurfa, Turkey; 4burakfe@gmail.com; 4 Department of Nuclear Medicine, Ankara University Faculty of Medicine, 06100 Ankara, Turkey; csoydal@yahoo.com

## Additional Affiliation

In published publication [1], there was an error regarding the affiliations for Bengu Dursun. Due to an oversight during the submission process, Bengu Dursun’s institutional affiliations were omitted. In addition to affiliation 2, the updated affiliations should include affiliation 1. The authors state that the scientific conclusions are unaffected. This correction was approved by the Academic Editor. The original publication has also been updated.
